# Population-based cohort study on the risk of malignancy in East Asian children with Juvenile idiopathic arthritis

**DOI:** 10.1186/1471-2407-14-634

**Published:** 2014-08-29

**Authors:** Victor C Kok, Jorng-Tzong Horng, Jing-Long Huang, Kuo-Wei Yeh, Jia-Jing Gau, Cheng-Wei Chang, Lai-Zhen Zhuang

**Affiliations:** Population-Health and Clinical Informatics Research Group, Department of Biomedical Informatics, Asia University Taiwan, Taichung, Taiwan; Division of Medical Oncology, Department of Internal Medicine, Kuang Tien General Hospital, Taichung, Taiwan; Department of Computer Science and Information Engineering, National Central University, Chungli, Taiwan; Division of Paediatric Allergy Asthma and Rheumatology, Department of Paediatrics, Chang Gung Memorial Hospital, Linkou, Taiwan; Chang Gung University College of Medicine, Taoyuan, Taiwan; Department of Information Management, Hsing Wu University, New Taipei City, Taiwan

**Keywords:** Arthritis, Juvenile rheumatoid (MeSH), Juvenile idiopathic arthritis, Neoplasms (MeSH), Risk (MeSH), Cohort studies (MeSH)

## Abstract

**Background:**

To investigate the association and magnitude of risk between JIA, its associated treatment and cancer development in Taiwanese children.

**Methods:**

Nationwide population-based 1:4 age- and gender-matched retrospective cohort study was designed using the National Health Insurance Research Database of Taiwan. A cohort of 2,892 children <16 years old with JIA was formed as well as a non-JIA cohort of 11,568 in year 2003 to 2005. They were followed up till a diagnosis of malignancy or up to 8 years until 2010. Relative risk (RR), incidence rate ratio (IRR), and adjusted hazard ratio (aHR) of developing malignancy were calculated.

**Results:**

The female to male ratio was 0.79:1. There were 3 cases of incident cancer in the “MTX use, biologics-naïve” group, only 1 in the anti-TNF biologics-containing group and 29 in the “both MTX- and biologics-naïve” group, in comparison, there were 50 cases of cancer in the non-JIA comparator group. During a 16114.16 patient-years follow-up, the RR and IRR for developing a malignancy in both methotrexate- and anti-tumor necrosis factor (TNF) biologics-naïve JIA children were 2.75 (95% confidence interval, 1.75 – 4.32) and 3.21 (2.01 – 5.05), respectively. For leukemia, the IRR was 7.38 (2.50 – 22.75); lymphoma, 8.30 (1.23 – 69.79); and soft tissue sarcoma, 11.07 (0.84 – 326.4). The IRR of other cancers was 2.08 (1.11 – 3.71). The aHR on cancer risk was 3.14 (1.98 – 4.98) in methotrexate- and biologics-naïve group. There were no statistically significant increased risk in JIA patients treated with methotrexate and/or anti-TNF biologics.

**Conclusions:**

Compared with children without JIA, children with JIA have 3-fold increase of risk on malignancy in East Asia. Seemingly neither methotrexate nor anti-TNF biologics increases the risk further.

## Background

Juvenile idiopathic arthritis (JIA) is a spectrum of several heterogeneous disorders manifested mainly as chronic inflammatory arthritis with no identifiable etiology with an onset before the age of 16 years. The older terms, juvenile rheumatoid arthritis (JRA) and juvenile chronic arthritis (JCA) have been replaced by the term juvenile idiopathic arthritis which is a more inclusive term than JRA [1].

Approximately 1 in every 1000 children worldwide has JIA [2, 3]. The best available annual incidence rate for JIA in the West is from a UK study which provided an estimate at 10/100,000 [4]. JIA appears to be less common in African-American and Asian populations [5]. A population-based study in Taiwan demonstrates that the prevalence of JIA in Taiwan is 3.7/100,000, which is higher than the prevalence in Japan, but lower than that in the West [6]. In addition to this ethnic difference in prevalence, previous studies revealed that the age of onset and gender differences in JIA are different between Caucasians and Asian children [6–8]. In our previously reported community-based cohort study of the clinical features in children with JIA in selected regions of Taiwan using the International League of Associations for Rheumatology system (ILAR) classification criteria, a remarkably high prevalence was found in the enthesitis-related arthritis (ERA; 37.4%) of the Chinese cohort, but a relatively low rate of uveitis (6.7%) as compared with previous reports on Western populations [9].

The first line treatment for JIA usually contains nonsteroidal antiinflammatory drugs (NSAIDs) and/or intraarticular glucocorticoid injection for controlling inflammation. Disease-modifying antirheumatic drugs (DMARDs), usually used in second line, are comprised of methotrexate (MTX) (conventional) and tumor necrosis factor (TNF-alpha) inhibitors (biologics) [10–14]. The criteria for escalation of therapy can be found in the paper published by the American College of Rheumatology elaborating the evidence-based recommendations for the treatment of JIA [15].

Two recent reports, one from the Canadian JIA registries maintained by paediatric rheumatology centers (JIA children = 1,834) and another from a Scottish nationwide population-based inpatient cohort (n = 349), showed no increase of risk for cancer over long-term follow-up [16, 17]. Nevertheless, three other recent studies notably showed increased risks of malignancy in patients with JIA [18–20].

Therefore, the aim of the present study was to estimate the magnitude of the risk of cancer in East Asian population of JIA and to examine the risk of cancer in children with JIA to the general population according to their treatment allocation.

## Methods

### Study design

**Figure 1:**
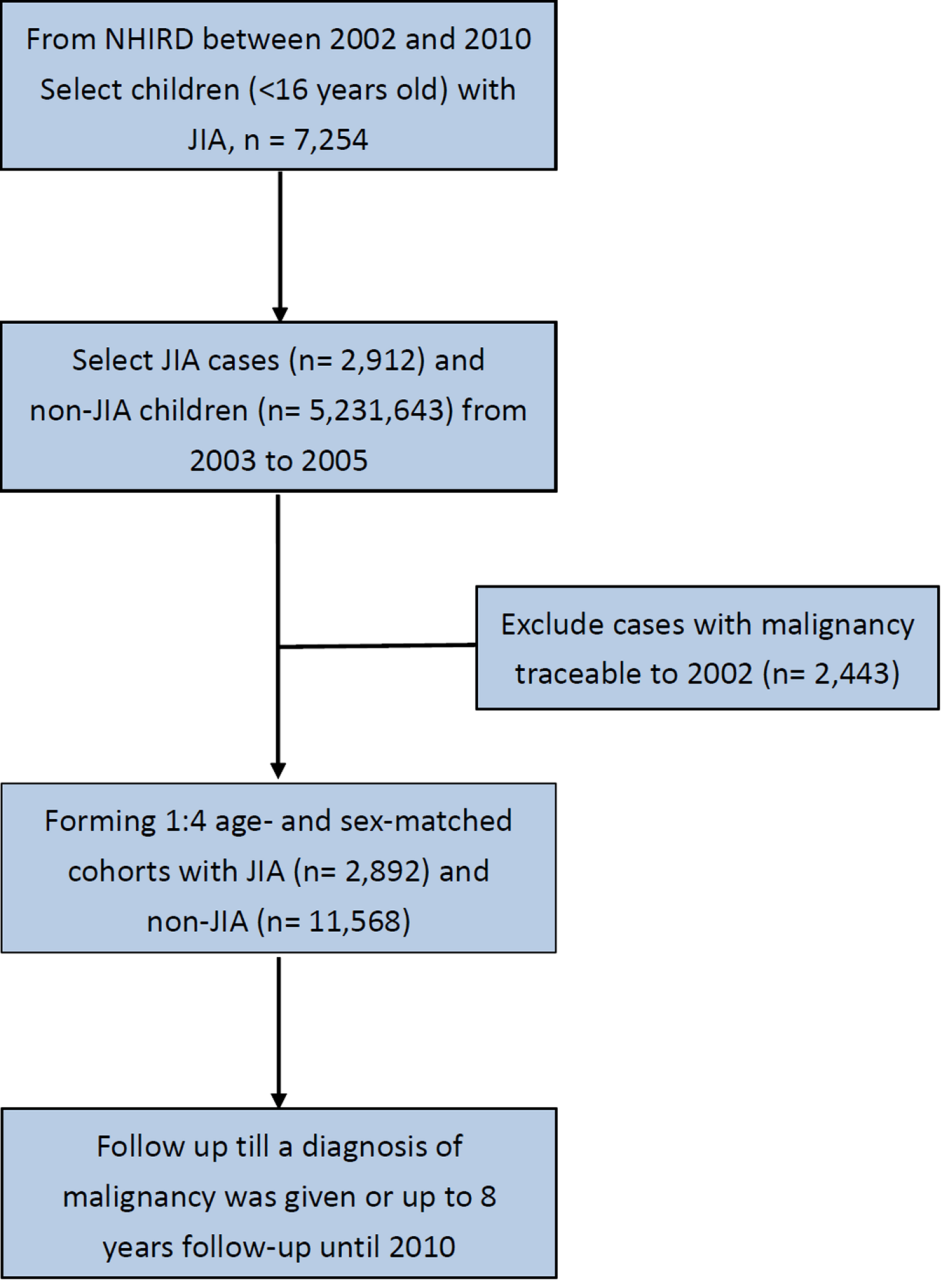
**Retrospective cohort study design and study flow chart.** NHIRD: National Health Insurance Research Database Taiwan; JIA: Juvenile idiopathic arthritis equivalent.

This study was designed as a population-based retrospective cohort study using data from a national administrative database with 1:4 gender- and age-matched comparators followed up to 8 years or until a diagnosis of malignancy was given. Figure 1 is a study flow chart demonstrating the design.

### Study population

The study population was targeted from the Taiwan National Health Insurance Research Database (NHIRD). The NHIRD has been described in-depth in our previous studies [6, 21–26]. In short, Taiwan National Health Insurance (NHI) program commenced on 1 March 1995. As of 2007, 98.4% of Taiwan’s population (22.96 million individuals) were enrolled in this program. The NHIRD provided to researches for academic research purposes contains a number of large computerized databases that incorporate registration files and relational original data on claims’ reimbursement. These data files are de-identified by scrambling the identification codes of both the individuals and medical facilities and access to the files including data-mining and data-manipulation shall strictly comply with the Taiwan Personal Information Protection Act.

### Case definition and definition of diseases/conditions

In Taiwan, paediatric rheumatologists prefer to use the ILAR classification criteria for making a diagnosis of JIA [27]. Grouping of ICD-9-CM codes can be mapped to the ILAR categories in patients under the age of 16: 714.2 (systemic arthritis), 714.30 + 714.31 (polyarthritis), 714.32 + 714.33 (pauciarthritis or oligoarthritis, persistent and extended), 720.0 alone or (720.0 + 714.0) or (720.0 + 714.3) (ERA), 696.0 (psoriatic arthritis).

A diagnosis of JIA in this study requires 3 components, age less than 16 years, at least 3 times of reimbursement claims made by a paediatric rheumatologist(s) or a paediatrician(s) using any of the abovementioned ICD-9-CM codes with the first and the third claim being 6 months apart.

### Study cohorts

From 2002 and 2010, we were able to identify all children less than 16 years old that diagnosed with JIA (n = 7,254). From these JIA children over a period of 9 years, we selected 2,912 children with JIA in years 2003 to 2005. Then children with JIA who had already been given a diagnosis of malignancy were excluded (n = 20). The criterion for this exclusion required only a single claim with a cancer ICD-9-CM code traceable back to at least one whole year in the medical record so that the accrual of patients were actually having no prevalent cancer. This left a group of children <16 years old with JIA but having no evidence of malignancy (n = 2,892) which became the JIA cohort for this study.

For forming a non-JIA comparator cohort, we did a 1:4 age- and sex-match for children with neither JIA nor cancer diagnosis traceable to the previous one year from the same period, 2003 to 2005. The index date for the comparator cohort was the earliest claim record. The non-JIA control group consisted 11,568 children.

### Grouping of JIA patients by treatment modalities

In the period of 2003 to 2005, there were 2 kinds of TNF-alpha inhibitor reimbursed by the NHI Taiwan for children with JIA, namely, etanercept and adalimumab. In the JIA cohort, three distinct groups could be separated by treatment allocation: biologics-naïve methotrexate group, anti-TNF biologics-containing group, and both methotrexate- and biologics-naïve group.

Treatment with TNF-alpha inhibitors was categorized as ever exposed versus never exposed. Once exposure to a TNF-alpha inhibitor was found, the status of “ever exposed” was maintained during the study follow-up. The person-time data for each therapeutic agent category did not include the person-time before the commencement of prescription of either MTX (in the biologics-naïve MTX group) or TNF-alpha inhibitors. Basically, the group being both methotrexate- and biologics-naïve can be regarded as children with JIA having no exposure to methotrexate or TNF-alpha inhibitors, thus the risk factor can be considered to be the diagnosis of JIA.

### Outcomes by identification of cancer cases

Each patient and control in the study was followed up to a maximum of 8 years through the NHIRD from accrual to identify whether the studied individual received a cancer diagnosis. The diagnostic accuracy of a specific kind of malignancy was confirmed by both a specific ICD-9-CM code within 140 to 208 given at least 3 times in different medical visits and inclusion into the Registry for Catastrophic Illness Patient Database (RCIPD). The index date was set on the date of the firstly detected code amongst three times claims using any one of the JIA ICD-9-CM codes.

### The scope and procedures of the search for the studies to be considered in the systematic review

We conducted a systematic review of the literature on the risk of cancer in children with JIA and whether the risk would further be increased after exposure to DMARDs. The criteria for inclusion of published studies for the review were cohort studies examining risk of cancer in terms of relative risks (RR), incidence rate ratio (IRR), or standardized incidence ratio (SIR), in children with JIA no matter receiving any kinds of treatment for JIA. We performed literature search in PubMed and DOAJ (the Directory of Open Access Journals) using medical subject headings (MeSH) terms combined with Boolean logics. The period of time covered by the literature search was from as early as the PubMed or DOAJ has covered until September of 2013. Table 1 outlines the result of this systematic review.

**Table 1:** **Outline of the results of the systematic literature review of the risk of malignancy in individuals with juvenile idiopathic arthritis**

Authors/Year published	Study setting/number of JIA patients	Outcome measure	Risk in JIA cohort	Risk in MTX exposed, biologics-naïve	Risk in TNF-alpha inhibitor exposed	Ref.
Beukelman T et al/2012	Using national Medicaid data/7,812	SIR	The SIR was 4.4 (95% CI, 1.8 – 9.0) for probable and highly probable incident malignancies. The unexposed (to both non-biologic and biologic DMARDs) group: the SIR = 6.9 (2.3 – 16).	SIR = 3.9 (95% CI 0.4 – 14)	Following any use of TNF inhibitors, SIR was 0 (95% CI 0 – 13).	20
Simard JF et al/2010	Nationwide cohort study through linkage with the Swedish Patient Register/5,296	RR	Subjects with JIA (biologics-naïve) identified in 1987 beyond (n = 5,296) was significantly associated with incident lymphoproliferative malignancies (RR 4.2, 95% CI 1.7–10.7) and cancers overall (RR 2.3, 95% CI 1.2–4.4).		-	18
Nordstrom BL et al/2012	Large claims database American cohort study/3,605	IR, SIR	The IRs of probable or highly probable cancer: 67.0 (95% CI, 1.3 – 132.5) cases/100,000 PY for biologics-naïve JIA and 23.2 (12.2 – 34.2) cases/100,000 PY for non-JIA. The JIA cohort had an elevated SIR of 4.0 (95% CI 2.6 – 6.0) above SEER rate.			19
Kok VC et al/this study	Nationwide population-based Retrospective cohort study/2,892	RR, IRR	The RR and IRR for developing a malignancy in JIA individuals who were both MTX- and anti-TNF biologics-naïve were 2.75 (95% CI, 1.75 – 4.32) and 3.21 (95% CI, 2.01 – 5.05), respectively, with the population attributable risk of 23.38% (95% CI, 10.56 – 36.21). For leukemia, the RR was 6.33 (2.30 – 17.44); lymphoma, 7.12 (1.42 – 35.61); and soft tissue sarcoma, 9.50 (1.24-72.46). The aHR on cancer risk was 3.14 (1.98 – 4.98) in MTX- and biologics-naïve group.	No further increase in risk	No further increase in risk	This study
Bernatsky S et al/2011	JIA registries at 3 Canadian pediatric rheumatology centers/1,834	SIR	Only one invasive cancer was identified in individuals with JIA observed for an average of 12.2 (SD 7.8) years (observation period: 1974 – 2006). SIR = 0.12 (95% CI, 0.0 – 0.7). The risk of invasive cancers overall is NOT increased. No further information on its relationship to drug exposure.			16
Thomas E et al/ 2000	Scottish nationwide population-based inpatient cohort/896	SIR	SIR in boys JIA with 2,647 person-years at risk: 1.29 (95% CI, 0.14–4.64); girls JIA with 3,940 person-years at risk, 0.83 (95% CI, 0.09–3.01)./*Not increased*		-	17

CI: confidence interval; IR: incidence rate; IRR: incidence rate ratio; JIA: juvenile idiopathic arthritis; MTX: methotrexate; RR: relative risk; SIR: standardized incidence ratio; anti-TNF: anti-tumor necrosis factor. Selected studies are arranged top down by decreasing number of patients in the JIA cohort.

### Statistical analysis

SAS statistical package (SAS System for Windows, version 8.2, SAS Institute, Cary, NC, USA), SPSSsoftware (version 17.0, IBM Inc., Chicago, Illinois, USA) and StatsDirect statistical software (StatsDirect Ltd, England, 2008) were used. The risk for cancer associated with JIA and various DMARDs exposure was evaluated and presented as a cumulative incidence ratio (RR) and HR in Cox regression. The RR which will not be influenced by immortal time was presented along with IRR. All statistical tests were two-sided. Values of *P* < 0.05 were considered statistically significant. Cox models providing adjusted HR (aHR) included age, gender, and treatment modality as covariates. HRs with a 95% confidence interval were calculated. The RR of cancer development were calculated and analyzed by Chi-square test (2×2). In addition, the impact of risk factors (how much disease burden is caused by certain risk factors) was also presented using the population attributable risk percent (PAR%) with 95% confidence interval in certain conditions.

This study has been approved by the Ethics Committee of Chang Gung Memorial Hospital Taiwan.

### Ethics statement

Because this study used only NHIRD data files that were de-identified by scrambling the identification codes of both individuals and medical facilities, this research fits the criteria for exemption from a full review by the Institution Review Board contained within the legal statements promulgated by the Ministry of Health and Welfare of Taiwan pursuant to Paragraph 1, Article 5 of the Human Subjects Research Act enacted on December 28, 2011. This study adhered to strict confidentiality guidelines that are in accordance with the regulations set forth by the Personal Information Protection Act of Taiwan, amended on May 26, 2010. The research was conducted in accordance with the Declaration of Helsinki as revised in 1989.

## Results

### Demographic characteristics of the JIA cohort

In the JIA cohort (n = 2,892), there were 1,618 boys and 1,274 girls. The female to male ratio was 0.79: 1. Most of the patients (n = 1,960, 67.8%) with JIA were in the age group of 11-15, 699 (24.2%) in age group 6-10, and 233 (8%) were in age group 0-5 (Table 2). Our results showed that the mean age of children with JIA assigned to the anti-TNF biologics group (mean, 10.54 years) were significantly younger than the other two groups, namely, biologics-naïve MTX group with mean age at 11.22 and both MTX- and biologics-naive group, 11.55 (Table 3).

**Table 2:** **Demographic data of JIA and non-JIA children in this cohort study**

		JIA (n = 2,892)	Non-JIA (n = 11,568)
Gender	Boys	1,618	6,472
	Girls	1,274	5,096
Female : male ratio		0.79 : 1	0.79 : 1
Age Group	0 - 5	233	932
	6 - 10	699	2,796
	11 - 15	1,960	7,840
Type of JIA by ILAR criteria			
	Polyarthritis	377	NA
	Pauciarthritis or oligoarthritis (persistent and extended)	136	NA
	Enthesitis-related arthritis	1221	NA
	Psoriatic arthritis	62	NA
	Systemic	6	NA
	Unclassifiable	1096	NA
Years of follow-up	Mean (SD)	6.40 (1.44)	7.78 (0.59)
	Person-years at risk	18,530.97	89,156.74

*Abbreviations:* DMARDs: Disease modifying anti-rheumatic drugs; ILAR: the International League of Associations for Rheumatology; JIA: juvenile idiopathic arthritis; MTX: methotrexate; NA: not applicable; SD: standard deviation; TNF: tumor necrosis factor.

It is noteworthy of the marked difference in male to female ratio in East Asian children as compared to Caucasians.

**Table 3:** **Exposure to methotrexate and/or an anti-TNF biologic in JIA patients and subsequent risk on malignancy development**

			JIA		Non-JIA	***P***-value
		MTX use, biologics-naive	Anti-TNF biologics-containing	Both MTX- and biologics-naive		
**Total**		344	112^Ϯ^	2436	11568	
**Gender**	Female	144	43	1087	5096	0.4849
	Male	200	69	1349	6472	
**Mean age (SD)**		11.22 (3.23)	10.54 (3.67)	11.55 (3.46)	10.94 (3.65)	<0.0001
**Age group (n)**	0 – 5	20	12	201	932	0.0054‡
	6 – 10	104	38	557	2796	
	11 – 15	220	62	1678	7840	
**Follow-up year, mean (SD)**		5.93 (2.08)	3.46 (1.82)	6.60 (1.10)	7.78 (0.59)	<0.0001
**Duration of drug exposure in year, mean (SD)**		2.97 (2.82)	3.05 (1.75)	NA	NA	0.78
**Malignancy**	Yes	3	1	29	50	<0.0001
	No	341	111	2407	11518	
**Person-years**		2030.08	386.73	16114.16	89156.74	
**Incidence rate ratio (95% CI)**		2.64 (0.65-7.53)	4.61 (0.23-23.61)	3.21 (2.01-5.05)	Ref	
**Relative Risk (95% CI)**		2.02 (0.67-6.04)	2.07 (0.36-11.49)	2.75 (1.75-4.32)*	Ref	
**Population attributable risk (95% CI)**		2.85% (-3.53-9.24)	1.01% (-2.82-4.84)	23.38% (10.56-36.21)	Ref	
**Leukemia**	Yes	2	0	8	6	
**IRR (95% CI)**		-	-	7.38 (2.50-22.75)*	Ref	
**Lymphoma**	Yes	0	0	3	2	
**IRR (95% CI)**		**-**	**-**	8.30 (1.23-69.79)*	Ref	
**Soft tissue sarcoma**	Yes	0	0	2	1	
**IRR (95% CI)**		-	-	11.07 (0.84-326.4)	Ref	
**Primary bone cancer**	Yes	0	0	1	1	
**IRR (95% CI)**		-	-	5.53 (0.14-215.8)	Ref	
**Other malignancy**	Yes	1	1	15	40	
**IRR (95% CI)**		-	-	2.08 (1.11-3.71)*	Ref	

CI: confidence interval; JIA: Juvenile idiopathic arthritis; MTX: methotrexate; NA: not applicable; Ref: reference; SD: standard deviation; TNF: tumor necrosis factor.

*statistically significant, P < 0.05.

^Ϯ^105 subjects (93.75%) previously or currently received MTX treatment.

‡: Kruskal-Wallis statistic.

Chi-square analysis-of-contingency table statistical method was for categorical data such as gender.

Poisson distribution and test-based methods are used to construct the confidence intervals for IRR.

In the JIA cohort, 344 patients, approximately 12%, were treated with methotrexate but were TNF-alpha inhibitor-naïve. One hundred and twelve patients (3.9%) required a TNF-alpha inhibitor to control their disease; 93.75% of them (n = 105) were previously or currently treated with methotrexate. Forty patients (1.4%) of JIA patients were treated with non-biologic DMARDs other than methotrexate which can possibly be sulfasalazine, hydroxychloroquine, leflunomide, azathioprine and/or cyclosporin in this country (Table 3).

The mean duration of follow-up for the JIA cohort was 6.40 (SD 1.44) years and 7.78 (SD 0.59) in non-JIA cohort.

### Risk of cancer

Compared to non-JIA children during 89156.74 patient-years at risk, children with JIA who were MTX- & biologics-naïve during 16114.16 patient-years at risk had significantly elevated RR for incident malignancy associated with JIA and IRR at 2.75 (95% CI 1.75 – 4.32) and 3.21 (95% CI 2.01 – 5.05), respectively. The cumulative incidence of cancer was significantly higher in MTX- & biologics-naïve JIA children group (1.19% vs. 0.43%, P <0.0001). The population attributable risk was 23.38% (95% CI, 10.56% – 36.21%) meaning among the general population, somewhere between 11% to 36% of the total risk for cancer was due to JIA status (Table 3).

With mean (SD) duration of follow-up of the JIA subgroups of “biologics-naïve methotrexate group” for 5.93 (2.08) years (2030.08 patient-years at risk), “anti-TNF biologics-containing” 3.46 (1.82) years (386.73 patient-years at risk), and “both methotrexate- and biologics-naïve” 6.60 (1.10) years (16114.16 patient-years at risk), we did not observe a statistically significant increased risk for cancer in JIA subgroups treated with methotrexate (RR 2.02; 95% CI, 0.67 – 6.04) or anti-TNF biologics (RR 2.07; 95% CI, 0.36 – 11.49) (Table 3). The mean duration of drug exposure in years for methotrexate group was 2.97 (2.82) and for anti-TNF inhibitor-containing group 3.05 (1.75).

The indirect standardized incidence rate (SIR) for all types of cancer in the non-JIA comparator cohort defined as ((Observed number of malignancies/Expected number of malignancies) × crude cancer incidence rate of the national population) was estimated. The crude cancer incidence rate of the national population aged from ten to twenty four years in the year 2008 was 17.10 per 100,000 as documented in the officially published Taiwan Cancer Registry, “Table Seven: Age distribution of cancer morphology by gender and primary anatomic sites 2008” (http://tcr.cph.ntu.edu.tw/main.php?Page=N2). The expected number of incident malignancies was three. Thus, the indirect SIR for all types of cancer in the non-JIA cohort equals 285 (50 / 3 × 17.1) per 100,000. It is higher than the nationwide average age-specific incidence rate for all types of cancer.

Based on the “both methotrexate- and biologics-naïve” subgroup of 2,436 patients with JIA having a mean follow up of 6.6 years, the incidence rate ratio for selected cancer types were calculated. There were increased IRR for leukemia at 7.38 (95% CI, 2.50 – 22.75), lymphoma 8.30 (95% CI, 1.23 – 69.79) and other malignancy mostly solid tumor 2.08 (95% CI, 1.11 – 3.71). However, there were no statistical increase of IRR in soft tissue sarcoma at 11.07 (95% CI, 0.84 – 326.4) and primary bone cancer 5.53 (0.14 – 215.8) (Table 3).

### Cox proportional hazard regression models

After adjustment for age and gender, the aHR for developing malignancy was 3.14 times higher (95% CI, 1.98 – 4.98, P <0.0001) in children with JIA who had not been exposed to either methotrexate or TNF-alpha inhibitors (Table 4). There were no further statistically significant increase of the risk in terms of aHR in children with JIA treated with either methotrexate whose aHR, 2.72 (95% CI, 0.85 – 8.73; P = 0.0932) or TNF-alpha inhibitors whose aHR, 6.05 (95% CI, 0.82 – 44.61; P = 0.0772) (Table 4). It merits mention that the anti-TNF biologics-containing group in the JIA cohort contained only 112 children.

**Table 4:** **Cox proportional hazard regression model showing the adjusted hazard ratio on the risk of developing malignancy in different groups of JIA patients by treatment and non-JIA patients**

	MTX use, biologics-naive	Anti-TNF biologics-containing	Both MTX- and biologics-naive	Non-JIA
aHR	2.72	6.05	3.14	1
95% CI	0.85 – 8.73	0.82 – 44.61	1.98 – 4.98	-
*P*-value	0.0932	0.0772	<0.0001	-

aHR: adjusted Hazard Ratio by gender and age; CI: confidence interval; JIA: juvenile idiopathic arthritis; MTX: methotrexate; TNF inhibitor: tumor necrosis factor.

## Discussion

Through this nationwide population-based retrospective cohort, we demonstrated that the incidence of malignancy was significantly increased approximately 3-fold in Taiwanese children with JIA. There were three cases of incident cancer in the “MTX use, biologics-naïve” group, only one in the anti-TNF biologics-containing group and twenty nine in the “both MTX- and biologics-naïve” group, in comparison, there were fifty cases of cancer in the non-JIA comparator group. The cancer types which were found to be statistically increased in terms of the IRR were leukemia (7-fold increase), lymphoma (8-fold) and all other malignancies (2-fold).

To the best of our knowledge, this cohort study presents the first best available estimate of the magnitude of risk for cancer in Asian JIA population. Our results in East Asian children in addition to three other prior large studies using claims database, National Medicaid data, or the Swedish Patient Register National Data, suggest that children with JIA per se is associated with a 2- to 4- fold increase of cancer risk, particularly lymphoproliferative malignancies (Table 1) [18–20]. However, our data did not reveal statistically significant further increase of incident malignancy in children with JIA after a mean (SD) duration of exposure of 2.97 (2.82) years to methotrexate and/or 3.05 (1.75) years to TNF-alpha inhibitors. Although anti-TNF biologics treatment are feared to increase the risk of malignancy particularly lymphoproliferative malignancy such as hepatosplenic T-cell lymphoma (HSTCL) which is the commonest reported subtype of all T-cell lymphoma, this phenomenon is still not yet confirmed in any published prospective cohort study [20, 28–33]. One of the explanations may be owing to the inherent susceptible risk already exists in children with JIA.

A recent detailed analysis of 100 cases of T-cell non-Hodgkin’s lymphoma (NHL) reported to the US FDA disclosed that 68% of the cases involved exposure to both a TNF-alpha inhibitor and an immunomodulator. Risk of T-cell NHL was higher with concomitant use of TNF-alpha inhibitor with thiopurines (95% CI 4.98-354.09; P < 0.0001) and thiopurines alone (95% CI 8.32-945.38; P < 0.0001) but not with TNF-alpha inhibitor use alone (95% CI 0.13-10.61; P = 1.00) [34]. Many of those reported cases that lead the FDA to place a black box warning on TNF-alpha inhibitors were in children with inflammatory bowel disease rather in JIA.

The female to male ratio in our JIA cohort was 0.79: 1. This ratio echoes other prior studies from Asia that in contrast to Caucasian population having female preponderance which ranges from 2: 1 to 3: 1 in most published series, Asian population of JIA has a relative male predominance. The explanation for this sex ratio difference is fairly clear that ERA, an entity of predominantly male and older age of onset, constituted 42% of the JIA cohort in this study. Our previous study mentioned earlier in the paper found that the proportion of ERA was 37.4% in the Taiwanese Chinese cohort [9]. In addition, other unique clinical features reported in Asian children are older age of onset, a lower antinuclear antibody (ANA) positivity rate, a lower chronic uveitis incidence and different subtypes of JIA on presentation [5, 6, 9].

In the JIA cohort, 15.5% ((344 + 105) / 2,892) of children required methotrexate for aggressive preservation of joint function and control of disease process. And only 3.9% of the entire JIA cohort were escalated to anti-TNF biologics therapy. In a published nationwide retrospective survey of 570 cases in Japan, 12.8% of their case series were treated with methotrexate [7]. This may reflect an overall real-world picture of the severity and treatment policy for JIA in East Asia.

### Strengths and limitations

Our study has certain strengths. Because participation in the NHI program is mandatory and all residents of Taiwan can assess a paediatrician rheumatologist without needing a referral, the referral bias is essentially avoided. Loss to follow-up is virtually non-exist because of the country-wide NHI coverage for medical care allowing continuous tracking of an enrollee during relocation or self-transferal. With a large number of age- and gender-matched non-JIA comparators, the cumulative incidence rate of cancer in the control group is considered accurate.

However, the findings of this study need to be interpreted in the context of certain potential limitations. First, many demographic variables were not available, such as family history of cancer, diet and environmental exposures. This is because individual identities are not available due to the de-identification of the individuals within the NHI databases. Second, the disease severity of JIA could not be obtained in this claims study. The risk of over-diagnosis resulted from solely using the ICD-9-CM codes for case identification is likely present due to inability to verify the diagnosis of JIA using the patients’ own medical records. We could not be 100% sure to exclude potentially exist undiagnosed JIA cases in the control group which may result in possible allocation bias. Finally, since this is not an incidence cohort for JIA and left censoring of disease and medication therapy cannot be excluded.

The possibility of a protopathic bias was not completely ruled out although the study design had deployed an exclusion criteria to exclude subjects with pre-existing cancer traceable back to at least one whole year in the medical record by ICD-9-CM codes.

## Conclusions

This nationwide population-based retrospective cohort study of the association between JIA by their treatment allocation and cancer risk with up to 8 years follow-up reveals that this East Asian children population with JIA is associated with a 3-fold increased risk of malignancy (IRR = 3.21 and adjusted HR = 3.14) and there is no statistically significant increased risk in these patients with exposure to methotrexate and/or TNF inhibitors. The risk for other solid tumors was statistically significantly increased by 2-fold.

## Authors’ information

JLH and KWY are professors of pediatric rheumatology and experts in juvenile idiopathic arthritis. VCK is a medical oncologist and medical informatician who is also teaching evidence-based medicine to junior medical staffs. JTH and CWC are experts in information engineering and big data management.
